# Sedative effects of *Arachis hypogaea* L. stem and leaf extracts on sleep-deprived rats

**DOI:** 10.3892/etm.2013.1182

**Published:** 2013-06-25

**Authors:** XIAOYAN ZU, ZHENYA ZHANG, GUANGQUAN XIONG, TAO LIAO, YU QIAO, YITING LI, SHENGRONG GENG, XIN LI

**Affiliations:** 1 Institute for Farm Products Processing and Nuclear-Agricultural Technology, Hubei Academy of Agricultural Sciences, Wuhan, Hubei 430064, P.R. China;; 2 Graduate School of Life and Environmental Sciences, University of Tsukuba, Tsukuba, Ibaraki 305-8572, Japan

**Keywords:** *Arachis hypogaea* L. stem and leaf, sedative effects, rat, sleep deprivation, linalool

## Abstract

*Arachis hypogaea* L. stem and leaf extracts (AHSLE) are reputed to aid sleep. The purpose of this study was to evaluate the sedative effects of AHSLE on sleep-deprived (SD) rats and the effect on energy system pathways. Furthermore, we analyzed the essential oil components of *Arachis hypogaea* L. stems and leaves (AHSL) to explain the sedative effects. AHSLE were obtained by extracting AHSL twice with water at 98°C for 3 h. Animal experiments were performed in the Laboratory Animal Resource Center, University of Tsukuba, Japan, and the levels of neurotransmitters were analyzed by high performance liquid chromatography (HPLC). The essential oil of the AHSL was obtained by simultaneous distillation and extraction (SDE) and analyzed by gas chromatography-mass spectrometry (GC-MS). Following treatment with AHSLE, the adenosine triphosphate (ATP) levels of the SD rats increased, which is a different effect from that previously observed in freely behaving rats. Adenosine (Ad) were not elevated by AHSLE uniformly throughout the brain, but accumulated in site-specific and time-prolonged manners. Following GC-MS analysis of the AHSL essential oil, a total of 37 compounds were identified; the major components were linalool (16.17%, which has sedative-like activity), *n*-hexadecanoic acid (16.42%), and 1-octen-3-ol (8.48%; a product of linalool decomposition). AHSLE affect the target neurotransmitters related to the rat circadian rhythms in specific brain regions, suggesting that AHSLE have the potential to increase sleep during the SD phase, and the sedative effects of AHSLE may be due to high levels of linalool and its decomposition products. AHSLE are potentially useful as sedatives or sleep aids in hypnotic therapy.

## Introduction

*Arachis hypogaea* L. stem and leaf extracts (AHSLE) have long had a reputation in China for easing various sleep disorders (1), and have been clinically validated by modern medicine (2,3). Previous studies have focused solely on the clinical effects, and few studies have considered their mechanisms and active components.

We have previously reported (4) that AHSLE results in the consumption of adenosine triphosphate (ATP) and the accumulation of adenosine (Ad) in corresponding areas of the brain, in freely behaving rats. These findings raised questions as to whether the effects of AHSLE on the behavior of rats differ between spontaneous sleeping and sleep-deprived (SD) rats, and whether the changes to the concentration of Ad induced by AHSLE occurred throughout the brains of the rats or were limited to one or more regions, such as those regions known to be important for sleep regulation. These questions prompted the experiments conducted in the current study.

Since in animals, circadian rhythms are affected by the nervous system of the brain and neurotransmitters (5–7), in the current study, we measured three neurotransmitters of ATP, adenosine diphosphate (ADP) and Ad, using high performance liquid chromatography (HPLC). We also analyzed the essential oil components of *Arachis hypogaea* L. stems and leaves (AHSL) by gas chromatography-mass spectrometry (GC-MS). The results were used to evaluate the hypnotic effects of AHSL and identify its mechanisms of action.

## Materials and methods

### Plants, AHSLE and reagents

AHSL were collected from the banks of Yezi Lake, Wuhan, Hubei, China in August 2011. The plant was authenticated by the Agriculture and Forestry Research Center of Tsukuba University, Japan. It was cleaned, air-dried and passed through a 40-mesh standard sieve for extraction. The AHSLE were obtained by a previously described method (4). The AHSL were extracted twice with 98°C water (3 h each time), followed by filtration to remove residue. Brown powders were obtained through rotary evaporation and freeze drying of the supernatant. ATP, ADP and Ad standards were purchased from Sigma Chemical Co. (St. Louis, MO, USA). Other chemicals used were purchased from Wako Pure Chemical Industries, Ltd. (Osaka, Japan).

### Animals

All animals and experiments were supported by the Laboratory Animal Resource Center of the University of Tsukuba, Japan. Male Sprague-Dawley rats (8 weeks old, 270±30 g) were housed at 25°C with 12 h light/dark cycles (light on 08:00, light off 20:00), and food and water *ad libitum*. All rat experiments were performed in a humane manner after receiving approval from the Institutional Animal Experiment Committee of Tsukuba University, and in accordance with the regulations for animal experiments and fundamental guidelines under the jurisdiction of the Japanese Ministry of Education, Culture, Sports, Science and Technology.

### Experimental protocols

In total, 34 rats were habituated in the animal lab for at least seven days, then randomly divided into two groups: the control group (water, n=16) and the AHSLE group [500 mg/kg body weight (BW), n=18]. Based on the method described previously (8) with certain modifications, the rats in the AHSLE group were randomly divided into three subgroups: SD-2.5 h (n=6), SD-5 h (n=6) and recovery (Rec)-3 h (n=6). All drug administration was conducted intragastrically (i.g.) for 14 days at a dose of 500 mg/kg BW before 8:00 a.m. (prior to the start of the light phase). On the 14th day, following drug administration, SD was achieved by the gentle handling method, as described previously (9). Rats were continuously inspected and SD was realized by tapping the cage lightly or touching the animal with a paintbrush. SD for 2.5 h or 5 h was started at 8:00 a.m. and performed under ‘lights-on’ conditions. The 3 h Rec started once the rats had been deprived of sleep for 5 h. The SD and Rec groups each had their own control group (n=5–6) of rats, which were treated under the same conditions.

Following the trials, the rats were anaesthetized with an intraperitoneal injection of urethane (200 mg/ml, 0.5 ml/100 g BW), and dissected rapidly to isolate the whole brains. To verify the differences in neurotransmitters at diverse brain regions, the entire brain was separated into three regions: the cerebrum, brainstem (midline involving thalamus and hypothalamus) and cerebellum. Considering the brain responses to nucleoside and nucleotide synthesis, the brain tissues were frozen immediately and stored at −80°C, prior to analyzing the target neurotransmitters.

### ATP, ADP and Ad analysis

ATP, ADP and Ad were analyzed by HPLC (JASCO International Co., Ltd, Tokyo, Japan). Rat brain samples were homogenized at 4°C with trichloroacetic acid (TCA) at a final concentration of 25% (v/v) and centrifuged to remove protein sediment. Supernatants were subsequently neutralized to pH 5–6 with NaOH, following filtration (0.45 *μ*m membrane). Analyses of the brain neurotransmitters, ATP, ADP and Ad, were performed according to previously reported methods (10) with minor modifications. The samples were analyzed by HPLC with a Capcell-Pak C18 column [4.6 mm internal diameter (I.D.) × 150 mm, particle size 5 *μ*m] at a flow rate of 1 ml/min and detected at 254 nm. The mobile phase consisted of a gradient mixture with solvent A (0.1 mol/l NH_4_H_2_PO_4_ solution) and B [20% (v/v) methanol (99.7%) in solvent A]. During testing, the gradient was as follows: 0 min, 0% B; 6–18 min, 35% B; 26 min, 0% B. The flow-rate was 1 ml/min with an injection volume of 20 *μ*l. The ATP, ADP and Ad standard were 100, 100 and 25 *μ*mol/l, respectively, in concentration and diluted by five gradients, respectively. In the brain samples, ATP and its metabolites were calculated by comparing peak areas and the appropriate standard curves.

### Essential oil extraction

Simultaneous distillation and extraction (SDE) was used to extract the essential oil from the AHSL. Petroleum ether and distilled water were used as low- and high-density solvents (contained 30 g AHSL/80 ml), respectively. The low- and high-density distillers for SDE were simultaneously heated by electric sets, so that the steam from the two distillers mixed with each other at the top of the glass instrument and subsequently divided into aqueous and petroleum ether layers. The petroleum ether layer with the essential oil from AHSL flowed to the low-density distiller and the aqueous layer flowed to the high-density distiller. The cyclical process was continued for 5–6 h, and three 30 g samples of AHSL were extracted by this method. The essential oil obtained was merged, following the removal of trace water by treatment with anhydrous Na_2_SO_4_. Following rotary evaporation, the essential oil in the petroleum ether was stored at −20°C.

### Chemical analysis of the essential oil

The essential oil in the petroleum ether was analyzed using an Agilent 7890 A chromatograph (Agilent Technologies Inc., Santa Clara, CA, USA) coupled with a 5975 network mass selective detector and equipped with a HP-5 capillary fused silica column (30 m × 0.25 mm I.D. × 0.25 *μ*m film thickness). The oven temperature was maintained at 80°C for 5 min, and then programmed to increase at a rate of 4°C/min to 280°C. Other operating conditions were as follows: carrier gas, He (99.999%) with a flow rate of 1 ml/min; injector temperature, 250°C; split ratio, 1:50; and injection volume, 1 *μ*m. The mass spectra were recorded at 70 eV with a mass range from m/z 20–500.

The linalool standard was at concentrations of 6.25, 12.5, 25, 50, 100 and 200 *μ*g/ml, with an internal standard of cyclohexanone with a final concentration of 100 *μ*g/ml. The linalool standard was analyzed by the method mentioned previously with the modification that the oven temperature was maintained at 80°C for 5 min and was then programmed to increase at 4°C/min to 160°C. The concentration of linalool in the essential oil was calculated by comparing the peak area and the appropriate standard curve.

### Statistical analysis

The data were analyzed using a two-tailed Student's t-test and the results are expressed as the means ± standard deviation. P<0.05, P<0.01 and P<0.001 are considered to indicate statistically significant differences.

## Results

### Brain ATP levels during SD and Rec sleep

**Figure 1.**
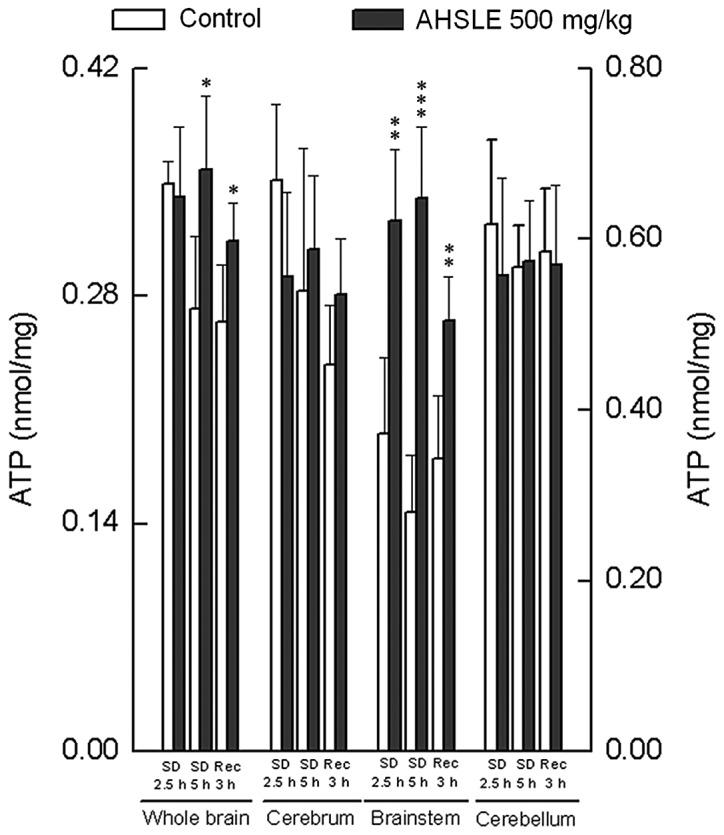
Adenosine triphosphate (ATP) concentrations in different brain regions during SD and Rec sleep. The principal y-axis represents the ATP level of the whole brain, cerebrum and brainstem. The secondary y-axis represents the ATP level of the cerebellum. Each column represents the mean with standard deviation. The significance of the effects of the treatment was assessed using the Student's t-test. ^*^P<0.05, ^**^P<0.01 and ^***^P<0.001 compared with the respective controls (open bars). SD, sleep-deprived; Rec, recovery.

It was demonstrated that in the AHSLE groups, brain ATP levels increased with SD time and decreased following 3 h Rec (Fig. 1). In the cerebrum and cerebellum, the ATP levels were reduced in the SD-2.5 h group, while they were increased in the SD-5 h group, compared with the respective controls. ATP levels were enhanced significantly (P<0.01) in the brainstems of the AHSLE-treated rats compared with the controls.

### Brain ADP levels during SD and Rec sleep

**Figure 2.**
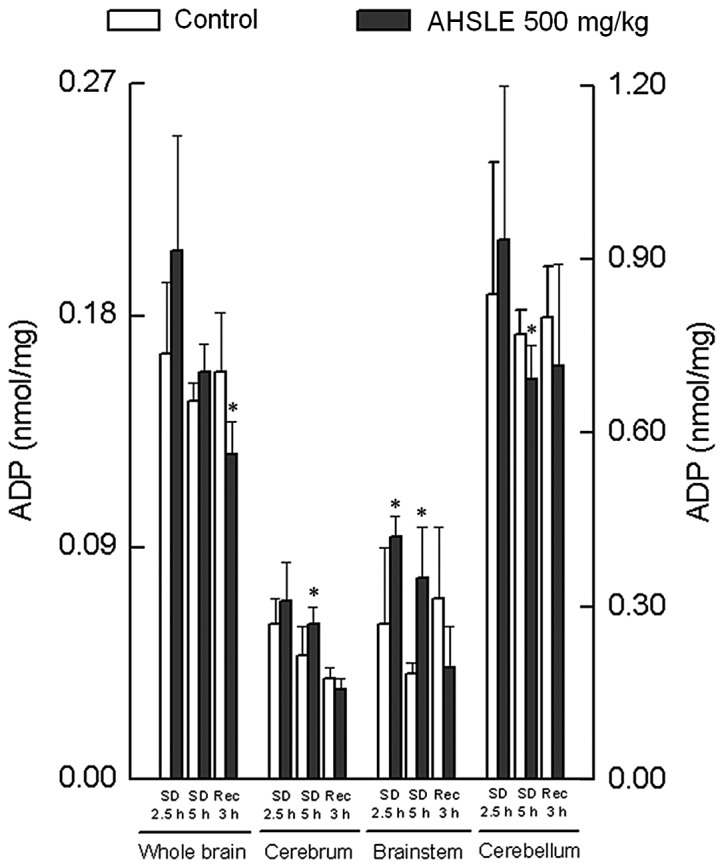
Adenosine diphosphate (ADP) concentrations in different brain regions during SD and Rec sleep. The principal y-axis represents the ADP level of the whole brain, cerebrum and brainstem. The secondary y-axis represents the ADP level of the cerebellum. Each column represents the mean with standard deviation. The significance of the effects of the treatment was assessed using the Student's t-test. ^*^P<0.05 compared with the respective controls (open bars). SD, sleep-deprived; Rec, recovery.

As the results (Fig. 2) demonstrated, the ADP levels decreased with increased SD time in the AHSLE and control groups. Following the administration of AHSLE, the ADP levels were enhanced during the SD phase compared with the respective control and the 3 h Rec did not increase the ADP values to normal levels.

### Brain Ad levels during SD and Rec sleep

**Figure 3.**
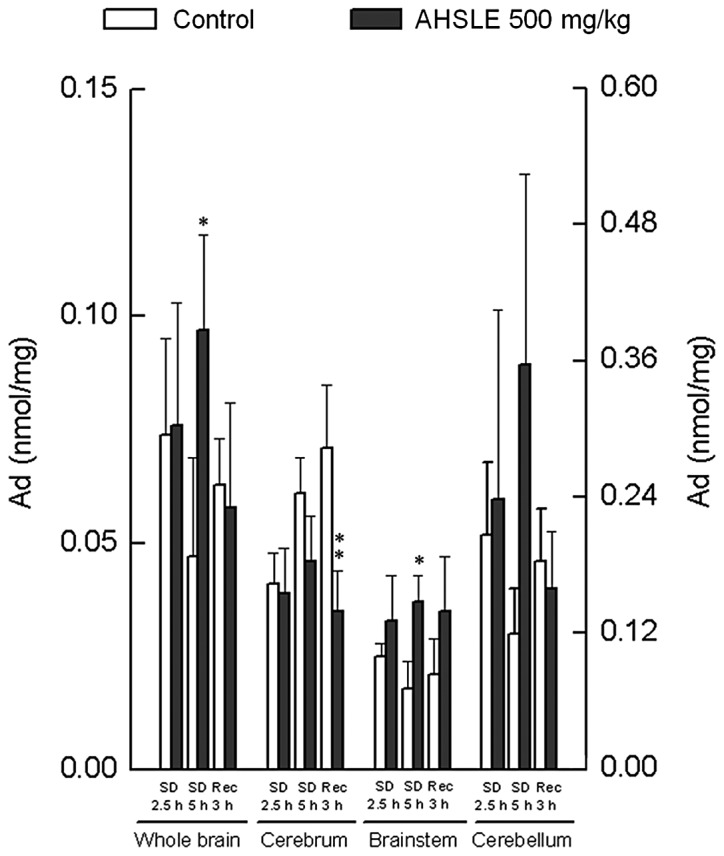
Adenosine (Ad) concentrations in different brain regions during SD and Rec sleep. The principal y-axis represents the Ad level of the whole brain, cerebrum, and brainstem. The secondary y-axis represents the Ad level of the cerebellum. Each column represents the mean with standard deviation. The significance of the effects of the treatment was assessed using the Student's t-test. ^*^P<0.05 and ^**^P<0.01 compared with the respective controls (open bars). SD, sleep-deprived; Rec, recovery.

According to the results (Fig. 3), the Ad values in the AHSLE groups increased with SD time in all brain regions in addition to the entire brain, and then reduced following 3 h Rec sleep. Following the administration of AHSLE, the Ad variation trend was similar to that of ATP; Ad accumulated in the brainstem and cerebellum during the SD phase. Contrasting results were observed in the control groups.

### Composition of AHSL essential oil

As can be seen in Table I, a total of 37 compounds were identified, and the major components were 3,7-dimethyl-1,6-octadien-3-ol (linalool, 16.17%), *n*-hexadecanoic acid (16.42%), 1-octen-3-ol (product of linalool decomposition, 8.48%,), 1,2,3-trimethylbenzene (6.56%), phytol (6.34%), 2-methoxy-4-vinylphenol (4.70%), 6,10,14-trimethyl-2-pentadecanone (3.96%), α,α,4-trimethyl-3 cyclohexene-1-methanol (3.80%), 1-ethyl-2-methylbenzene (3.74%), *cis*-linalool oxide (3.54%), 1,3,5-trimethylbenzene (2.94%) and benzeneacetaldehyde (2.81%; Table I). These 12 major components accounted for 79.46% of the yield, while other identified constituents represented <2%. The linalool standard curve obtained was Y=2926.4 × −16702, R^2^=0.9997. According to the peak area and standard curve, the linalool concentration in petroleum ether was ∼10.415 mg/ml, and yield was ∼578.611 mg/kg AHSL.

## Discussion

ATP is the direct source of energy for cellular activity. ADP may be converted into ATP or degraded to Ad (11). Energy depletion may increase the need to sleep (12). The ATP results for the control group (Fig. 1) indicate that brain levels of ATP decreased with increasing SD time in the whole brain and 3 h of Rec sleep was insufficient to replenish the energy lost. In contrast to the SD-2.5 h control group, the ATP levels in the AHSLE-2.5 h group increased in the brainstem while they decreased in the cerebrum and cerebellum, which revealed the preferential provision of energy to the central area and indicated that energy deficiency in the cerebrum and cerebellum may establish the need to sleep. When AHSLE were administered, the ATP levels of the SD rats increased, which is different from the effect previously observed in freely behaving rats (4); this may be due to state-related changes to the stress reactions of the rats.

Ad is a crucial neurotransmitter in the sleep system of the brain (13); it accumulates in the brain and has been proposed as an important factor for homeostatic sleep (14). In AHSL-treated SD rats, the observed increases in the concentrations of Ad in all regions of the brain reflected the bio-energetic stress of the animals. Furthermore, 3 h of Rec sleep was sufficient to reduce the sleep propensity signal (Ad) to the levels observed during normal wakefulness, following the administration of AHSLE.

The purine nucleoside, Ad, has been reported to be a candidate sleep factor, as systemic injections of Ad were observed to promote sleep and reduce wakefulness (15). The concentrations of Ad have also been reported to increase with increased metabolism and neural activity (16,17). In the current study, in SD rats, Ad accumulated as a product of increased metabolism/neural activity, to inhibit neural activity in various brain regions and was then consumed during the recovery of sleep.

The current study demonstrated that, during sleep deprivation, Ad accumulated in a site-specific and time-prolonged manner. In the brain stem and cerebellum, Ad levels in the AHSLE groups increased steadily and significantly during the SD phase, compared with basal and SD-2.5 h levels. By contrast, the cerebrum did not demonstrate an accumulation of Ad during the SD phase following the administration of AHSLE, indicating that, during prolonged wakefulness, Ad levels are not uniformly elevated by AHSLE throughout the brain. These findings support the hypothesis that an increase in the need to sleep enhances the release of cortical Ad. It indicates that the cerebellum and brainstem are critical sites of Ad action. Thus, these data revealed that during sleep deprivation, AHSLE stimulates Ad accumulation selectively in the cerebellum and, to a lesser extent, in the brainstem.

Regarding the effective components, we analyzed the composition of the essential oil of AHSL according to a previously described method (18). Naturally occurring linalool may be transformed into a number of derivatives that are valuable to the flavour and fragrance industries (19,20), and has demonstrated antidepressant- and sedative-like activity (21). The sedative effect of linalool-rich essential oils is suggested to provoke a reduction in neuronal excitability (22). Our analysis of the essential oil components of AHSL indicated a high linalool content, which may be the source of the sedative effects.

In conclusion, this site-specific accumulation of Ad during sleep deprivation indicates the physiological regulatory effect of AHSLE. Cumulative evidence suggests that AHSLE increases the levels of Ad in the brain during sleep deprivation, which has the potential to increase sleep. The sedative effects of AHSL may be attributed to their high linalool content.

## Figures and Tables

**Table I.** Components identified in the essential oil of AHSL by GC-MS analysis.

Number	Retention time (min)	Compound	Peak area (×10^5^)	Relative area^a^ (%)
1	2.310	(E)-2-Hexenal	0.190	0.52
2	2.503	*p*-Xylene	0.620	1.69
3	3.740	1-Ethyl-2-methylbenzene	1.371	3.74
4	3.984	1-Octen-3-ol	3.109	8.48
5	4.285	2-Pentylfuran	0.231	0.63
6	4.405	1,2,3-Trimethylbenzene	2.403	6.56
7	5.148	1,3,5-Trimethylbenzene	1.076	2.94
8	5.290	Limonene	0.093	0.25
9	5.738	Benzeneacetaldehyde	1.031	2.81
10	6.590	*cis*-Linalool oxide	1.297	3.54
11	7.004	1-Ethyl-2,4-dimethylbenzene	0.082	0.22
12	7.435	3,7-Dimethyl-1,6-octadien-3-ol (linalool)	5.929	16.17
13	7.594	3,7-Dimethyl-1,5,7-octatrien-3-ol	0.343	0.94
14	10.591	α,α-4-trimethyl-3-cyclohexene-1-methanol	1.393	3.80
15	10.789	(−)-Myrtenol	0.248	0.68
16	11.936	2,7-Dimethyl-2,6-octadien-1-ol	0.180	0.49
17	12.855	(E)-3,7-Dimethyl-2,6-octadien-1-ol	0.600	1.64
18	14.910	2-Methoxy-4-vinylphenol	1.721	4.70
19	17.231	1-(2,6,6-Trimethyl-1,3-cyclohexadien-1-yl)-2-buten-1-one	0.245	0.67
20	20.466	N-(4-methoxyphenyl)-2-propenamide	0.390	1.06
21	21.771	5,6,7,7a-Tetrahydro-4,4,7a-trimethyl-2(4H)-benzofuranone	0.203	0.55
22	22.827	(E)-3,7,11-Trimethyl-1,6,10-dodecatrien-3-ol	0.377	1.03
23	23.854	Trichloroacetic acid, 3-methylbutyl ester	0.549	1.43
24	29.092	Sulfurous acid, 2-propyl tridecyl ester	0.159	0.43
25	29.319	2,6,10-Trimethyl-dodecane	0.180	0.49
26	30.148	6,10,14-Trimethyl-2-pentadecanone	1.451	3.96
27	30.676	Phthalic acid, isobutyl octadecyl ester	0.171	0.47
28	31.334	Tritetracontane	0.391	1.07
29	31.777	6,10,14-Trimethyl-(E,E)-5,9,13-pentadecatrien-2-one	0.225	0.61
30	31.919	Hexadecanoic acid, methyl ester	0.463	1.26
31	32.724	*n*-Hexadecanoic acid	6.017	16.42
32	35.256	11,14-Eicosadienoic acid methyl ester	0.358	0.98
33	35.375	12-Methyl-E,E-2,13-octadecadien-1-ol	0.513	1.40
34	35.613	Phytol	2.324	6.34
35	38.905	3-Ethyl-5-(2-ethylbutyl)octadecane	0.302	0.83
36	40.568	Heptacosane	0.220	0.60
37	42.157	Eicosane	0.204	0.56
		Total	36.659	99.96

a Relative area (%) is peak area relative to the total peak area with the exception of solvent, internal standard and impurity peaks.

## References

[1] Wang QC, Xu J, Shi M (2001). Clinical observation of preparation from the branch and leaf of peanut in treating insomnia. Shanghai J Trad Chin Med.

[2] Hu PF, Fan RP, Li YP, Pang CY (2001). Studies on pharmacological action of Luohuashengzhiye extract. Chin Trad Pat Med.

[3] Wang YF, Li HF, Xu YF, Zhang YL, Xu DS, Xiao LM, Li XM (2001). Clinical confirmation of preparation from the branch and leaf of peanut in treating insomnia. Shanghai J Trad Chin Med.

[4] Zu XY, Zhang ZY, Liu JQ, Hu HH, Xing GQ, Zhang Y, Guan D (2010). Sedative effects of peanut (*Arachis hypogaea* L) leaf aqueous extracts on brain ATP AMP, adenosine and glutamate/GABA of rats. J Biomed Sci Eng.

[5] Sherin JE, Shiromani PJ, McCarley RW, Saper CB (1996). Activation of ventrolateral preoptic neurons during sleep. Science.

[6] Gaus SE, Strecker RE, Tate BA, Parker RA, Saper CB (2002). Ventrolateral preoptic nucleus contains sleep-active galaninergic neurons in multiple mammalian species. Neuroscience.

[7] Sherin JE, Elmquist JK, Torrealba F, Saper CB (1998). Innervation of histaminergic tuberomammillary neurons by GABAergic and galaninergic neurons in the ventrolateral preoptic nucleus of the rat. J Neurosci.

[8] Wigren HK, Schepens M, Matto V, Stenberg D, Porkka-Heiskanen T (2007). Glutamatergic stimulation of the basal forebrain elevates extracellular adenosine and increases the subsequent sleep. Neuroscience.

[9] Franken P, Dijk DJ, Tobler I, Borbély AA (1991). Sleep deprivation in rats: effects on EEG power spectra, vigilance states, and cortical temperature. Am J Physiol.

[10] Schweinsberg PD, Loo TL (1980). Simultaneous analysis of ATP, ADP, AMP, and other purines in human erythrocytes by high-performance liquid chromatography. J Chromatogr.

[11] Dworak M, Diel P, Voss S, Hollmann WK, Strüder H (2007). Intense exercise increases adenosine concentrations in rat brain: implications for a homeostatic sleep drive. Neuroscience.

[12] Thakkar MM, Engemann SC, Walsh KM, Sahota PK (2008). Adenosine and the homeostatic control of sleep: effects of A1 receptor blockade in the perifornical lateral hypothalamus on sleep-wakefulness. Neuroscience.

[13] Huang ZL, Urade Y, Hayaishi O (2007). Prostaglandins and adenosine in the regulation of sleep and wakefulness. Curr Opin Pharmacol.

[14] Kalinchuk AV, Urrila AS, Alanko L, Heiskanen S, Wigren HK, Suomela M, Stenberg D, Porkka-Heiskanen T (2003). Local energy depletion in the basal forebrain increases sleep. Eur J Neurosci.

[15] Ticho SR, Radulovacki M (1991). Role of adenosine in sleep and temperature regulation in the preoptic area of rats. Pharmacol Biochem Behav.

[16] Radulovacki M (1985). Role of adenosine in sleep in rats. Rev Clin Basic Pharm.

[17] Van Wylen DG, Park TS, Rubio R, Berne RM (1986). Increases in cerebral interstitial fluid adenosine concentration during hypoxia local potassium infusion and ischemia. J Cereb Blood Flow Metab.

[18] Jalali Heravi M, Sereshti H (2007). Determination of essential oil components of *Artemisia haussknechtii* Boiss. using simultaneous hydrodistillation-static headspace liquid phase microextractiongas chromatography mass spectrometry. J Chromatogr A.

[19] Letizia CS, Cocchiara J, Lalko J, Api AM (2003). Fragrance material review on linalool. Food Chem Toxicol.

[20] Simić A, Soković MD, Ristić M, Grujić-Jovanović S, Vukojević J, Marin PD (2004). The chemical composition of some *Lauraceae* essential oils and their antifungal activities. Phytother Res.

[21] Guzmán-Gutiérrez SL, Gómez-Cansino R, García-Zebadúa JC, Jiménez-Pérez NC, Reyes-Chilpa R (2012). Antidepressant activity of *Litsea glaucescens* essential oil: identification of β-pinene and linalool as active principles. J Ethnopharmacol.

[22] de Almeida RN, Araújo DA, Gonçalves JC, Montenegro FC, de Sousa DP, Leite JR, Mattei R, Benedito MA, de Carvalho JG, Cruz JS, Maia JG (2009). Rosewood oil induces sedation and inhibits compound action potential in rodents. J Ethnopharmacol.

